# Machine learning identified MDK score has prognostic value for idiopathic pulmonary fibrosis based on integrated bulk and single cell expression data

**DOI:** 10.3389/fgene.2023.1246983

**Published:** 2023-11-24

**Authors:** Shichen Zhang, Lanlan Zhang, Lu Wang, Hongqiu Wang, Jiaxin Wu, Haoyang Cai, Chunheng Mo, Jian Yang

**Affiliations:** ^1^ Center of Growth, Metabolism, and Aging, Key Laboratory of Bio-Resources and Eco-Environment, College of Life Sciences, Sichuan University, Chengdu, China; ^2^ State Key Laboratory of Respiratory Health and Multimorbidity, Department of Respiratory and Critical Care Medicine, West China Hospital, Sichuan University, Chengdu, China; ^3^ Systems Hub, The Hong Kong University of Science and Technology (Guangzhou), Guangzhou, China; ^4^ Key Laboratory of Birth Defects and Related Diseases of Women and Children of MOE, State Key Laboratory of Biotherapy, West China Second University Hospital, Sichuan University, Chengdu, China

**Keywords:** idiopathic pulmonary fibrosis, machine learning, midkine, single cell sequencing, integrated analysis

## Abstract

Idiopathic pulmonary fibrosis (IPF) is a progressive and fatal lung disease that poses a significant challenge to medical professionals due to its increasing incidence and prevalence coupled with the limited understanding of its underlying molecular mechanisms. In this study, we employed a novel approach by integrating five expression datasets from bulk tissue with single-cell datasets; they underwent pseudotime trajectory analysis, switch gene selection, and cell communication analysis. Utilizing the prognostic information derived from the GSE47460 dataset, we identified 22 differentially expressed switch genes that were correlated with clinical indicators as important genes. Among these genes, we found that the midkine (MDK) gene has the potential to serve as a marker of Idiopathic pulmonary fibrosis because its cellular communicating genes are differentially expressed in the epithelial cells. We then utilized midkine and its cellular communication-related genes to calculate the midkine score. Machine learning models were further constructed through midkine and related genes to predict Idiopathic pulmonary fibrosis disease through the bulk gene expression datasets. The midkine score demonstrated a correlation with clinical indexes, and the machine learning model achieved an AUC of 0.94 and 0.86 in the Idiopathic pulmonary fibrosis classification task based on lung tissue samples and peripheral blood mononuclear cell samples, respectively. Our findings offer valuable insights into the pathogenesis of Idiopathic pulmonary fibrosis, providing new therapeutic directions and target genes for further investigation.

## 1 Introduction

Idiopathic pulmonary fibrosis (IPF) is a chronic and progressive lung disease characterized by the accumulation of scar tissue in the lungs, leading to difficulty breathing and chronic respiratory failure (Martinez et al., 2017; Chanda et al., 2019). The disease primarily affects older adults and is associated with high mortality rates, with a median survival of 3–5 years if untreated. The exact cause of IPF is not yet fully understood, though it is believed to be a result of a combination of genetic susceptibility and environmental exposures such as smoking, air pollution, and viral infections (Martinez et al., 2017). Currently, treatment options for IPF are limited, and there is still much to discover about its underlying mechanisms and potential therapeutic targets.

Some studies have focused on several key pathways involved in IPF pathogenesis, including epithelial-mesenchymal transition (EMT) and extracellular matrix (ECM) dysregulation (Chanda et al., 2019; Peng et al., 2020). In response to environmental triggers, immune cells such as macrophages and T cells are activated, leading to the release of pro-inflammatory cytokines and chemokines (Lee et al., 2021; Tanner et al., 2023). This activation results in the recruitment and activation of fibroblasts, which contribute to excessive ECM deposition and scarring in the lungs. EMT is a process in which epithelial cells lose their characteristic properties and acquire mesenchymal characteristics, enabling them to migrate and differentiate into other cell types. In IPF, EMT contributes to the accumulation of activated fibroblasts and myofibroblasts, which play a major role in ECM remodeling and fibrosis. ECM dysregulation is a hallmark of IPF and is characterized by excessive deposition and remodeling of ECM proteins such as collagen, fibronectin, and elastin. Understanding the complex interactions between these pathways and identifying potential therapeutic targets are major areas of focus in current IPF research (Martinez et al., 2017; Chanda et al., 2019).

The single-cell technique is a high-throughput analytical technique that enables gene expression profiling of individual cells, allowing for the detection of subtypes and functional differences between different cells, identification of rare cell types, and discovery of disease-related key genes and pathways at the cellular level (Sklavenitis-Pistofidis et al., 2021). Moreover, single-cell studies have also made significant contributions to the understanding of the pathogenesis of IPF. For instance, Morse et al. revealed an increase in fibroblasts, basal cells, ciliated cells, and club cells in IPF. They also identified macrophages expressing high levels of SPP1 and MERTK, which contribute significantly to lung fibrosis (Morse et al., 2019). Adams et al. discovered a unique basal cell population in IPF that expresses markers associated with basal cells, epithelial cells, mesenchymal cells, aging, and development. These findings suggest that the appearance of this cell population may be related to EMT in IPF patients (Peng et al., 2020). Additionally, Kobayashi et al. focused on the pre-alveolar type-1 transitional cell state (PATS) and found that markers of stratifin (*SFN*), tumor protein p63 (*TP63*), keratin 17 (*KRT17*), and *TP63* are co-expressed with collagen type I alpha 1 chain (*COL1A1*) in highly fibrotic cells, resulting in an aberrant elongated shape of the PATS cells (Kobayashi et al., 2020). Despite the progress made in understanding IPF through these studies, the specific pathogenesis of IPF, as well as the underlying causes of EMT and ECM formation in IPF, remain unclear and require further investigation.

In this study, we integrated five bulk gene expressing datasets and performed a comprehensive analysis with single-cell RNA sequencing (scRNA-seq) data results. We identified several differentially expressed genes that have clinical relevance and provided new insights into pathogenic factors, such as ECM and EMT, that are involved in IPF. Specifically, we constructed an SVM classifier for the MDK gene and related communication genes, achieving high accuracy in both lung tissue and peripheral blood sequencing datasets. These findings offer new directions for future research into the pathogenesis of IPF.

## 2 Results

### 2.1 Integrated bulk gene expression datasets identified consistently differentially expressed genes

**FIGURE 1:**
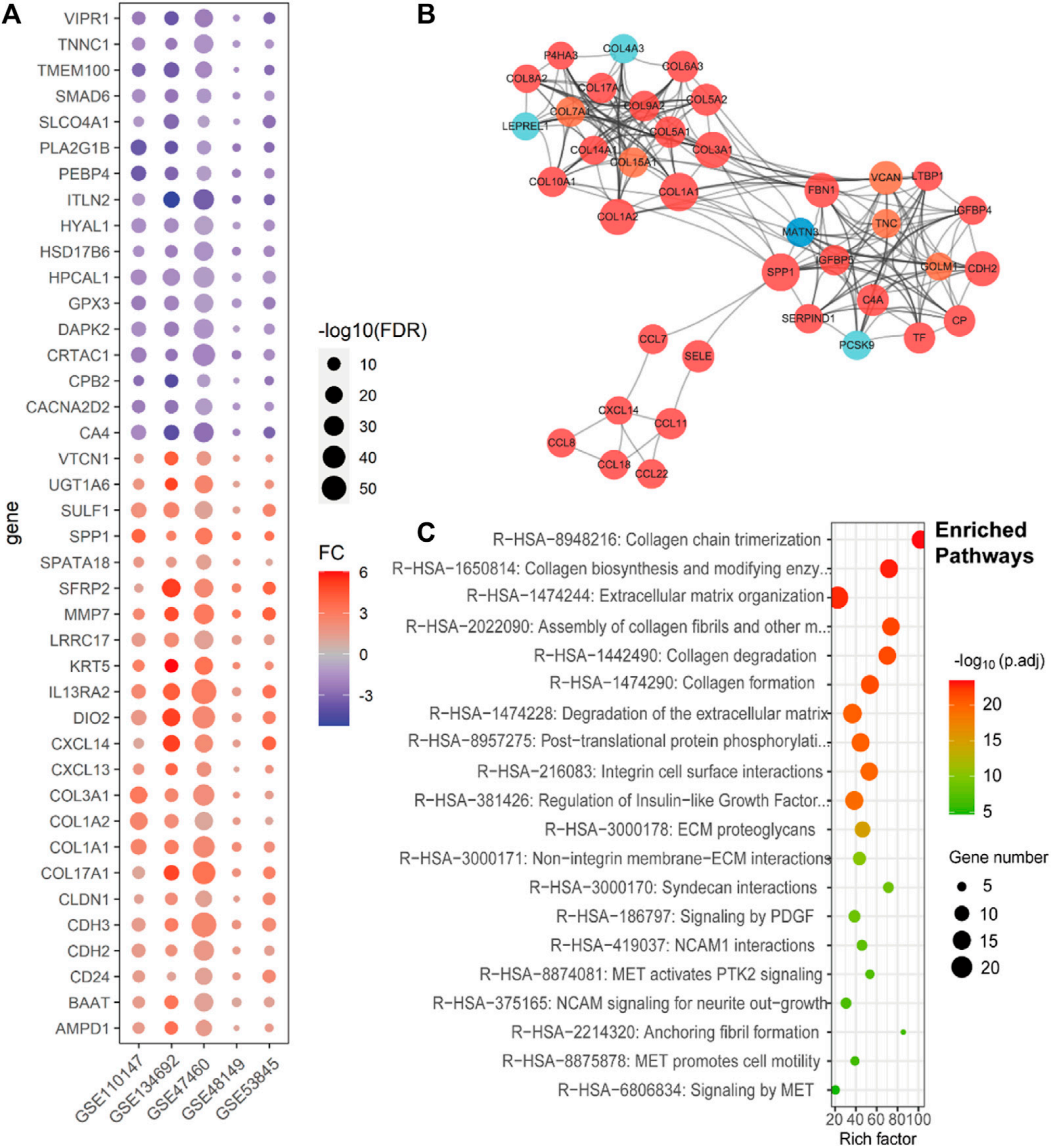
Consistent differentially expressed genes from bulk gene expression datasets, their subnetwork, and enriched pathways of IPF. **(A)**. Dot plot shows the fold change and *p*-values of consistent DEGs in all five lung tissue bulk gene expression datasets (GSE110147, GSE134692, GSE47460, GSE48149, and GSE53845). **(B)**. The sub-network from the protein–protein interactions network (PPI network), which includes the genes related to ECM of the IPF, with the red ones representing high expression in IPF, orange ones representing both high expression and being clinically related in IPF, light blue ones representing low expression in IPF, and deep blue one representing both low expression and being clinically related in IPF. **(C)**. The Reactomes enrich pathways related to the subnet of Figure 1B.

Following acquisition of the bulk gene expression datasets, we conducted an analysis and identified a total of 1215 differentially expressed genes (DEGs). Among these DEGs, 745 were upregulated in more than two datasets, and 23 genes were consistently upregulated in all five datasets (Figure 1A, Supplementary Figure S1). Notably, the upregulated genes, such as *MDK*, tetraspanin 1 (*TSPAN1*), *COL1A1*, and collagen type I alpha 2 chain (*COL1A2*), were found to be enriched in extracellular matrix-related pathways, cytokines and cytokine receptor pathways, and collagen binding pathways (Supplementary Figures S1A, S1B). On the other hand, the downregulated genes were primarily enriched in G protein-coupled receptor (GPCR) signaling and cytokine binding (Supplementary Figures S1C, S1D).

To elucidate the interplay among the DEGs and provide insight into their biological functions in IPF, we conducted protein–protein interaction (PPI) network analysis using the STRING database, which enabled identification of subnetworks. Notably, we identified a subnet consisting of the *COL1A1* and *COL1A2* genes, which showed significant enrichment in extracellular matrix (ECM)-related pathways (Figure 1B), predominantly comprising upregulated genes. This finding corroborated previous research, highlighting the pivotal role of ECM in IPF pathogenesis (Figure 1C).

### 2.2 Single-cell atlas of IPF lung tissues reveals the roles of different cell types in IPF

**FIGURE 2:**
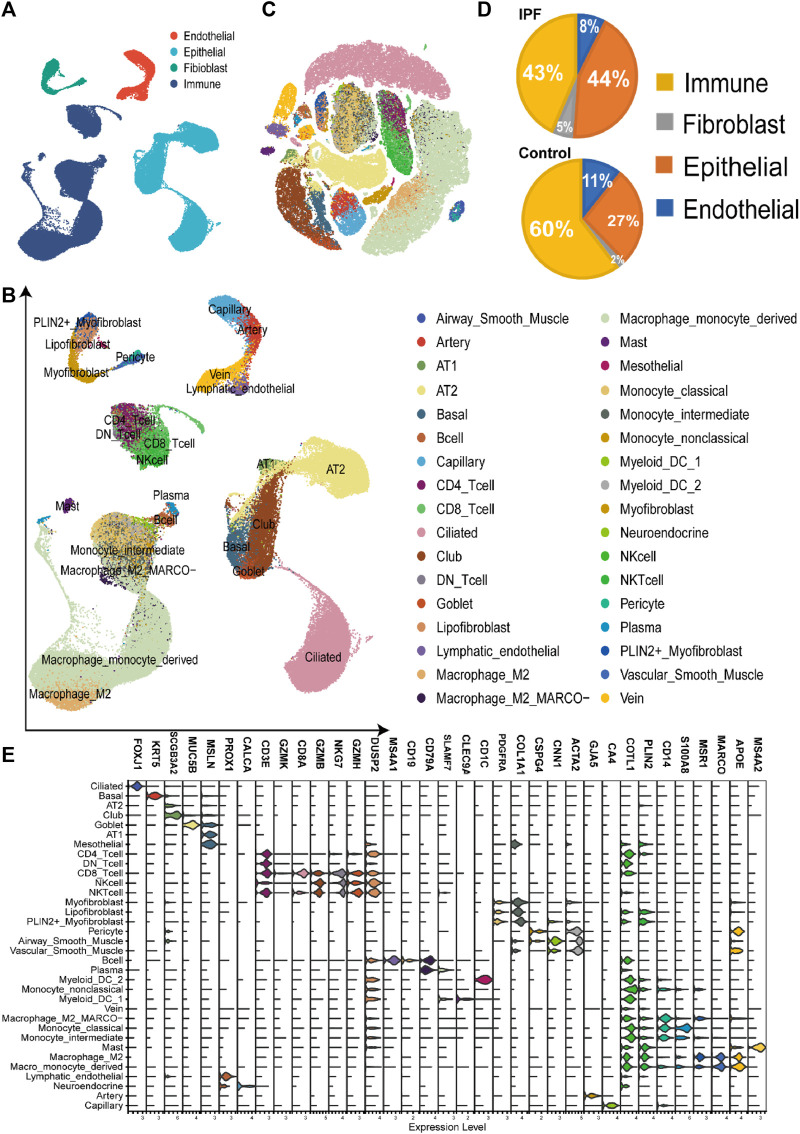
Construction of single-cell RNA-seq atlas of the lung tissue from IPF **(A)**. The UMAP plot of four main cell types in single-cell sequencing data, including endothelial cells, immune cells, epithelial cells, and fibroblasts. **(B)**. The UMAP plot of the single-cell sequencing data, including 83752 cells of 34 cell types from the lung tissue. **(C)**. The TSNE plot with the label of different cell types, which have the same label as Figure 2B. **(D)**. The cell proportion of four main types in different groups, revealing the high proportion of fibroblasts and epithelial cells in the IPF group. **(E)**. The volcano plot of the cell markers in different types of cells in the single-cell sequencing data.

During the single-cell RNA sequencing (scRNA-seq) process, we initially selected IPF and normal samples from the GSE135893 dataset, specifically targeting IPF and normal control samples. We excluded samples diagnosed with interstitial lung disease (ILD) from the dataset (Habermann et al., 2020a). After discarding empty droplets, doublet cells, and dead cells, we ultimately identified a total of 54,151 cells from 12 IPF samples and 29,601 cells from 10 normal samples. We annotated these cells as belonging to four primary groups based on the marker genes and unsupervised clustering: fibroblasts, endothelial cells, epithelial cells, and immune cells (Figure 2A). Subsequently, each primary group was further divided into specific cell types, including 16 types of immune cells, 4 types of endothelial cells, 7 types of epithelial cells, and 7 types of fibroblasts (Figures 2B, C).

We further performed cell proportion analysis, pseudotime analysis, switch gene selection, and cell communication analysis on the primary cell types. For immune cells, we identified 124 downregulated and 182 upregulated DEGs (Supplementary Table S2), with a subset of 8 downregulated and 12 upregulated DEGs observed in the bulk gene expression data. Similarly, for endothelial cells, we detected 327 downregulated and 270 upregulated DEGs (Supplementary Table S2), among which 79 downregulated and 14 upregulated DEGs were also identified in the bulk gene expression data.

**FIGURE 3:**
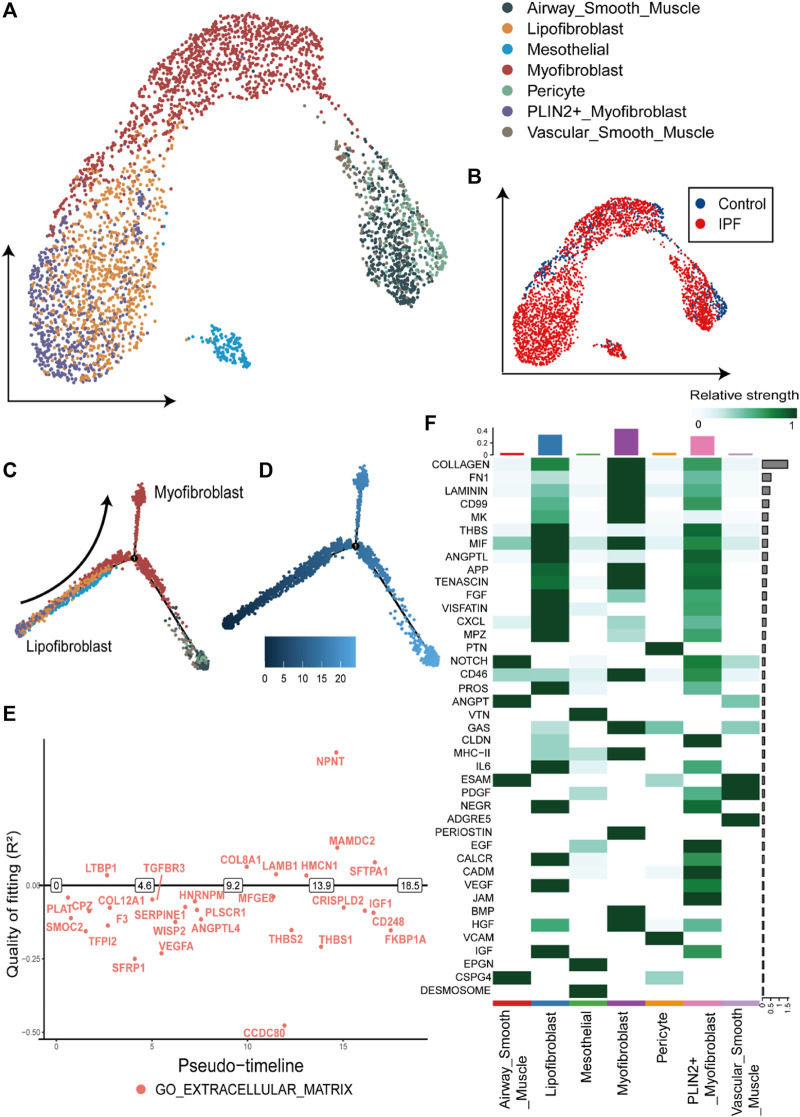
Overview and pseudotime results of fibroblasts from single-cell RNA-seq dataset **(A)**. The UMAP plot of the fibroblasts in single cell sequencing data, containing seven subtypes of fibroblasts. **(B)**. The UMAP plot with the labels of different groups, with the blue representing the control group and red the IPF group. **(C)**. Pseudotime trajectory plot of fibroblast calculated by monocle2. The trace from left to right reveals the trace from lipofibroblasts to myofibroblasts. **(D)**. The pseudotime of the cell development trajectory plot in Figure 3C. **(E)**. The switch DEGs of the GO: extracellular matrix pathway in the trace from lipofibroblasts to myofibroblasts. **(F)**. The strength of the cell-to-cell communication pathways in fibroblasts from the IPF group. The collagen-related communication shows the strongest communication.

Within fibroblasts, we identified 334 downregulated DEGs and 569 upregulated DEGs (Supplementary Table S2), with 24 downregulated and 68 upregulated DEGs overlapping with the DEGs from bulk gene expression data. The proportion of fibroblasts has increased from 2% in the control group to 5% in the IPF group (Figure 2D). Notably, a subgroup of fibroblast exhibiting high expression of gene markers for both myofibroblasts [*COL1A1*, actin alpha 2, smooth muscle (*ACTA2*)] and lipofibroblasts [*COL1A1*, perilipin 2 (*PLIN2*)] was identified and classified as *PLIN2* ^+^ myofibroblasts (Figures 2B,C; Figure 3A). Most of the myofibroblasts and lipofibroblasts were derived from IPF patients (Figure 3B, Supplementary Figure S5F). We analyzed the pseudotime trajectory from the lipofibroblasts to myofibroblasts in IPF (Figures 3C,D), and identified 17 transcription factors and 34 surface proteins, many of which were related to ECM pathways (Supplementary Figure S3, Figure 3E). Additionally, cell communication analysis revealed strong communication in ECM-related pathways, particularly in collagen signaling (Figure 3F), which are critical components of ECM, and has been implicated in IPF (Chanda et al., 2019; Hamanaka et al., 2019).

**FIGURE 4:**
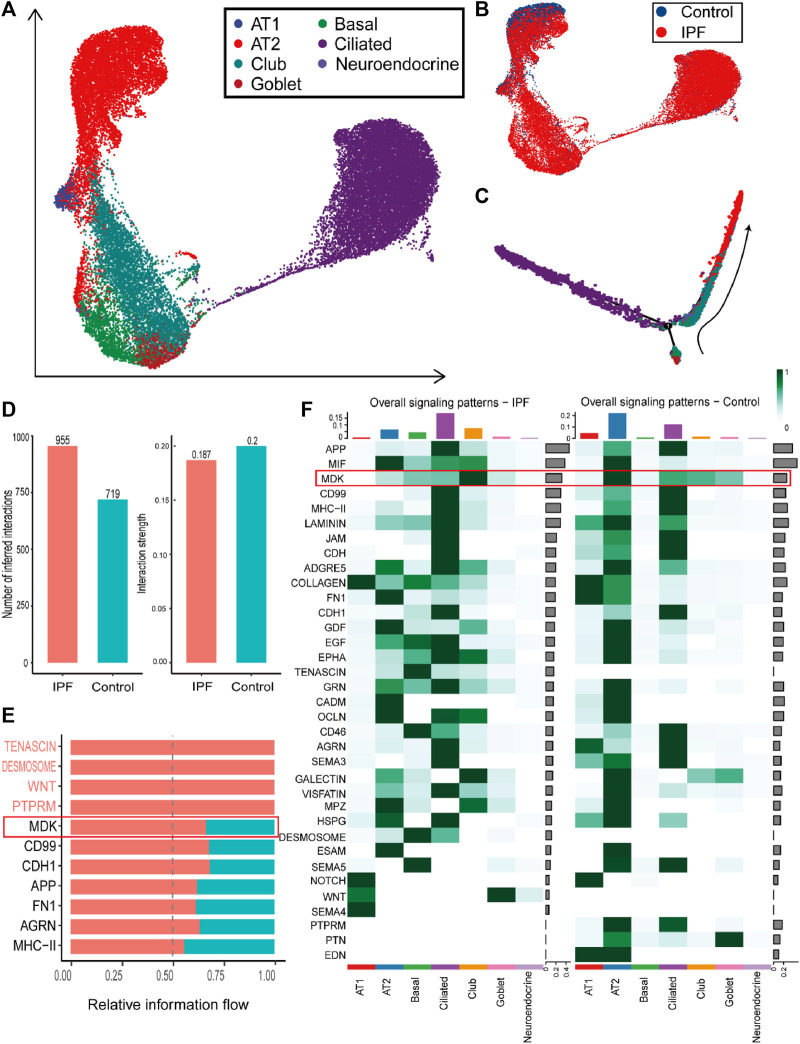
Overview and communication results of epithelial cells from single-cell RNA-seq dataset **(A)**. UMAP plot of the epithelial cells in single-cell sequencing data containing seven subtypes of epithelial cells. **(B)**. UMAP plot with the labels of different groups of epithelial cells, with blue representing the control group and red the IPF group. **(C)**. Pseudotime trajectory plot of epithelial cells calculated by monocle2. The trace from beneath to right up reveals the trace from basal cells to AT2 and AT1 cells. **(D)**. The number of inferred cell-to-cell interactions (left) and the interaction strength (right) in epithelial cells. **(E)**. The upregulated pathways in the communication of epithelial cells from the IPF group. **(F)**. The comparison of overall signaling patterns in the IPF and control groups of epithelial cells.

The most significant difference between IPF and control groups was found in the epithelial cells. There were 107 downregulated DEGs and 163 upregulated DEGs identified in both the single-cell dataset and bulk gene expression (Supplementary Table S1 and Supplementary Table S2). The proportion of the epithelial cells in IPF patients was higher (44%) compared to the control group (27%) (Figure 2D, Supplementary Figure S5G). Specifically, epithelial cells from IPF samples were predominantly ciliated and club cells, while normal epithelial cells were primarily composed of alveolar type 2 progenitor (AT2) cells (Figures 4A, B). To gain a better understanding of the transition of epithelial cells, we analyzed the pseudotime trajectory from basal cells to AT2 cells and identified switch genes in this trace from control and IPF groups (Figure 4C). In the IPF group, a total of 1241 genes were identified as switch genes. Among these genes, there were 83 differentially expressed genes with absolute log2 fold change (|log2FC|) > 0.58. Additionally, we found 87 surface proteins, including MDK, *TSPAN1*, and serpin family F member 1 (*SERPINF1*), as well as 37 transcription factors, such as nuclear receptor 4A 1 (*NR4A1*) (Supplementary Table S3). In the control group, 1198 genes were identified as switch genes, including 74 differentially expressed genes, 79 surface proteins (including *MDK*, TIMP metallopeptidase inhibitor 1 (*TIMP1*), and *TSPAN1*), and 39 transcription factors (including *NR4A1*) (Supplementary Table S3). Among these genes, 28 were identified as distinct switch genes between IPF and control groups, with 8 exhibiting differential expression. For the common switch genes, by intersecting with DEGs in bulk gene expression datasets and single cell datasets, specific genes such as *MDK* and *TSPAN1* are highlighted.

Furthermore, cell communication analysis revealed more interactions and similar strengths in the IPF group compared to the control group (Figure 4D). In particular, cell communication that was more expressed in IPF epithelial cells was mainly concentrated in MDK, CD99, and other pathways (Figure 4E). Besides, ECM-related cell communication was found to be increased in basal cells and ciliated cells but decreased in AT2 and AT1 cells (Figure 4F). Notably, we identified a potentially important gene, *MDK*, based on multiple lines of evidence. Firstly, the expression of the *MDK* gene was found to be upregulated in both bulk datasets and epithelial cells. Secondly, MDK was identified as a switch gene in both the IPF and Control groups. Thirdly, MDK-related communication pathway genes showed differential expression between the IPF and Control group. The related midkine pathway also exhibited significant differences between the IPF and control groups (Figure 4F), suggesting that MDK plays a major role in the progression of IPF. These findings provide novel insights into the underlying mechanisms of IPF pathogenesis and offer potential targets for therapeutic intervention.

### 2.3 Clinical indexes correlation analysis in GSE47460 identified the clinically related genes of IPF

To evaluate the clinical relevance of the differentially expressed genes and switch genes, we analyzed the dataset GSE47460. We calculated the Pearson’s correlation coefficient (PCC) between gene expression data and various clinical indicators, such as pre- and post-bronchodilator Forced Expiratory Volume (FEV1), pre- and post-bronchodilator Forced Vital Capacity (FVC), and diffusing capacity of the lungs for carbon monoxide (DLCO). Among the differentially expressed genes identified from five lung tissue gene expression datasets, we found 143 genes that showed moderate correlation with clinical indicators, including 79 upregulated genes and 64 downregulated genes (|PCC| > 0.4). Specifically, upregulated genes were negatively correlated with clinical indicators, whereas downregulated genes showed the opposite trend (Supplementary Figure S4). Additionally, enriched pathway analysis revealed that the upregulated genes associated with clinical relevance were primarily involved in ECM and GPCR binding-related pathways (Supplementary Table S2). These findings provide valuable insights into the link between gene expression and clinical indicators in IPF patients.

In order to further screen the related genes of IPF disease, we defined the genes satisfying the following conditions as important genes: 1. DEGs obtained from bulk gene expression datasets, 2. DEGs of different cell types from single-cell sequencing, 3. switch genes in main cell types, and 4. genes related to clinical indicators. This led to the identification of 22 genes (Supplementary Table S5). Among these genes, caveolin 1 (*CAV1*), insulin-like growth factor (*IGF1*), and *TSPAN1* have previously been reported as potential markers of IPF (Lin et al., 2019; Liu et al., 2019; Hernandez et al., 2020). For other genes, glutathione peroxidase 3 (*GPX3*) was identified as a switch gene in both endothelial and immune cells and as a DEG in all five gene expression datasets. Furthermore, the *MDK* gene, as previously mentioned, may play an important role in the development of IPF in epithelial cells through the MDK-related pathway and the MDK-TSPAN1 ligand-receptor pair.

### 2.4 Regulation of *MDK* genes in epithelial and endothelial cells of IPF

**FIGURE 5:**
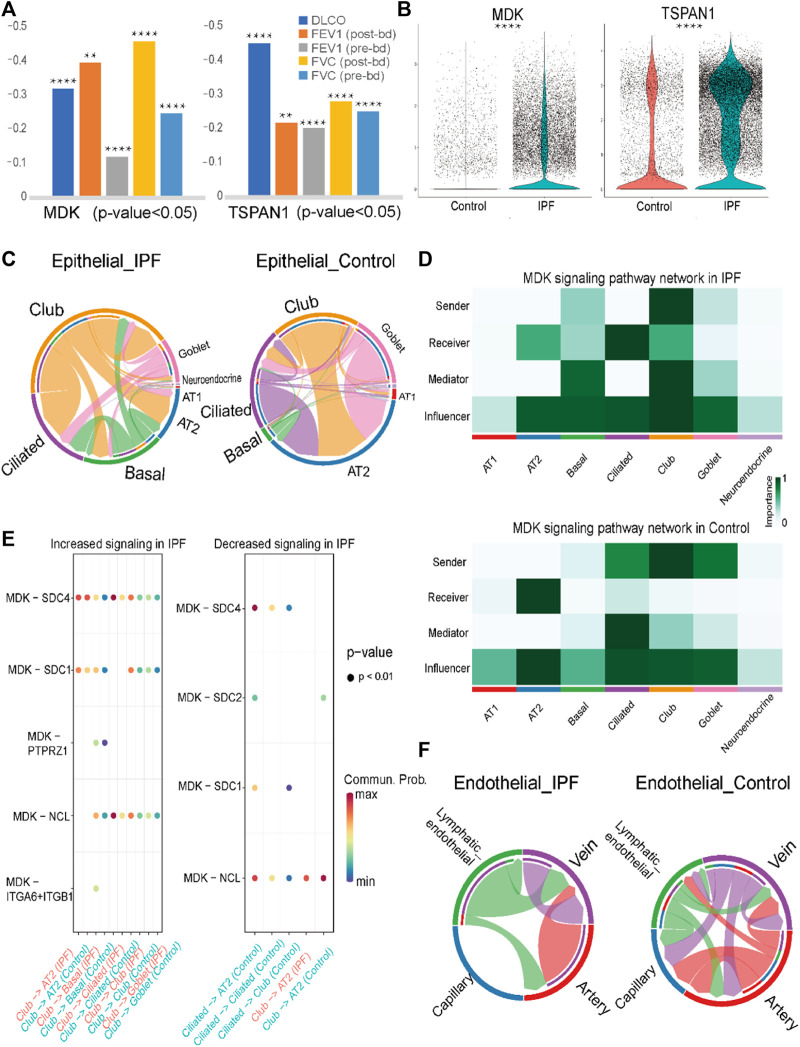
Analysis results of MK signaling pathway and prediction result of lung tissue datasets **(A)**. Pearson coefficient of MDK and TSPAN1 gene in five clinical indexes in GSE47460. **(B)**. Violin plot of MDK and TSPAN1 in the epithelial cells, with the red representing the control group and the blue representing the IPF group. **(C)**. Chord chart of cell-to-cell communication of the MK signaling pathway in epithelial cells, with the left one being IPF and the right one the control group. **(D)**. The MK signaling pathway network in the epithelial cells, with the upper one being the IPF group and the lower one being the control group. **(E)**. The bubble plot of increasing and decreasing signaling ligand–receptor pairs in IPF, with high communication of club cells being seen in IPF and low communication in AT2 cells in IPF. **(F)**. Chord chart of MK signaling pathway network in endothelial cells. Statistical analysis was performed to verify the Pearson correlation, or the two-sample *t*-test was used for comparisons between two groups. *p < 0.05, **p < 0.01, ***p < 0.001, ****p < 0.0001.

Through integrative analysis of bulk gene expression and single-cell RNA sequencing data, we identified MDK as an important gene in IPF. MDK was upregulated in three gene expression datasets, similar to TSPAN1 expression, which is another important gene and composed ligand-receptor pair with MDK. Both MDK and TSPAN1 were significantly correlated with clinical indicators. Specifically, MDK expression showed a negative correlation with FEV indices and DLCO index (post-bronchodilator FEV: coefficient = −0.47; pre-bronchodilator FEV: coefficient = −0.4; DLCO: coefficient = −0.32), while TSPAN1 was negatively correlated with the DLCO index (coefficient = −0.45) (Figure 5A). Moreover, MDK was highly expressed in both endothelial and epithelial cells in the single-cell RNA sequencing data, whereas TSPAN1 exhibited high expression specifically in epithelial cells (Figure 5B). Furthermore, MDK was identified as a switch gene in the cell trajectory analysis from basal cells to AT2 cells in both IPF and control groups together with TSPAN1 (Supplementary Table S3).

Through cell communication analysis, we observed that the MDK gene exhibited a high degree of communication with club cells and ciliated cells in IPF group. In contrast, AT2 cells showed increased communication in the control group (Figure 5C, Supplementary Figure S3A). Specifically, in the IPF group, club cells were identified as the senders in the MDK communication pathway, with ciliated cells and club cells acting as the main receivers. Other cells, including AT2 cells and basal cells, acted as mediators and influencers in the communication process (Figure 5D). In contrast, in addition to club cells, the senders in the control group were also comprised of ciliated cells and goblet cells, with only AT2 cells serving as the receivers (Figure 5D). Other cells, such as basal cells, club cells, and ciliated cells, were relatively reduced in the MDK signaling pathway network. These findings highlight the complex interactions involved in MDK-mediated cell communication in the context of IPF.

To elucidate the mechanisms underlying the transition of ciliated cells from senders to receivers in the IPF group, we conducted an analysis of the ligand-receptor pairs in the IPF and control groups. The results revealed that MDK-nucleolin (NCL), MDK- syndecan 1 (SDC1), MDK-SDC2, and MDK-SDC4 were involved in signaling from ciliated cells to AT2 cells in the control group, while no such signal was detected in the IPF group. Moreover, the ligand-receptor pairs MDK-NCL and MDK-SDC4 were involved in signaling from ciliated cells to ciliated cells or club cells in the control group, whereas in the IPF group, MDK-SDC4 and MDK-NCL exhibited higher expression in basal cells, club cells, and goblet cells compared to ciliated cells. These findings may be related to the abnormal expression patterns observed in the epithelial cells of IPF. Additionally, other differences in cell communication were mainly observed between club and AT2 cells (Figure 5E).

In addition to its role in epithelial cells, MDK has also been identified as a DEG in endothelial cells, prompting us to conduct an analysis of its cellular communication. The results revealed that in IPF, lymphatic endothelial cells and vein cells primarily functioned as senders, with vein cells acting as the main receivers. In contrast, in the control group, the role of lymphatic endothelial cells was diminished, and the communication was predominantly observed in the artery and capillary endothelial cells (Figure 5F, Supplementary Figure S3B, C). The ligand-receptor pairs involved in the MDK signaling pathway in endothelial cells are mainly comprised of MDK-NCL and MDK-[integrin subunit alpha 6 (ITGA6) + integrin subunit beta 1 (ITGB1)] (Supplementary Figure S3D). Taken together with the findings from our analysis of epithelial cells, these results highlight the differential expression pattern and cellular communication mechanisms of MDK and their potential implications for disease pathogenesis.

### 2.5 SVM models accurately classify the IPF using MDK and its communication genes in both lung tissue and PBMC datasets

In the previous section, we identified MDK as a crucial gene involved in the pathogenesis of IPF. The regulation of MDK is primarily mediated by two potential pathways: the MDK-TSPAN1 ligand–receptor pair and the MK signaling pathway in epithelial cells. We hypothesized that differences in the MK signaling pathway network in ciliated cells, club cells, and AT2 cells may play a critical role in the development of IPF. To investigate whether MDK and related receptors can serve as markers for IPF, we constructed a machine learning model utilizing gene expression data from both lung tissue and PBMC samples. Our aim is to examine the diagnostic potential of MDK and its associated genes in identifying patients with IPF.

**FIGURE 6:**
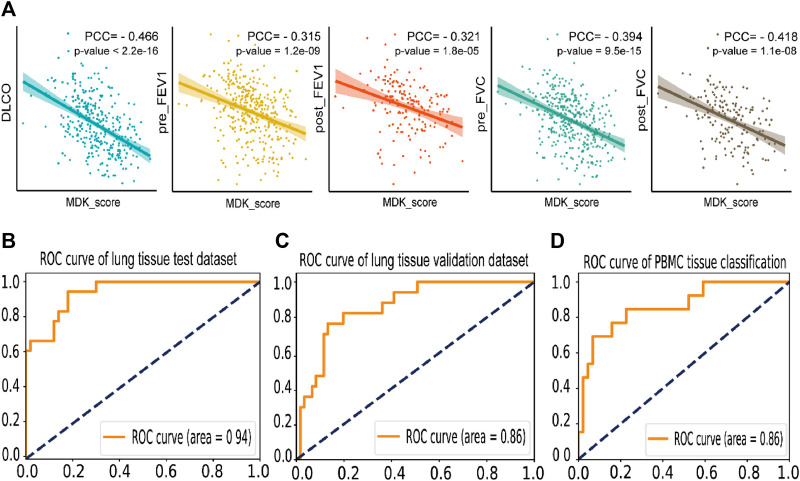
Prediction and correlation analysis result of MK score and MDK related genes **(A)**. Dot plot of the Pearson coefficient of MK score in five clinical indexes in GSE47460, labeling the value of the Pearson coefficient and the *p*-value of the result. **(B)**. The ROC curve of the IPF disease classification test dataset by SVM model based on the expression of MDK and related genes in lung tissue bulk gene expression data, with an AUC = 0.94. **(C)**. The ROC curve of the individual validation dataset of lung tissue by SVM based on the expression of MDK and related genes. **(D)**. The ROC curve of the IPF disease classification by SVM model based on the expression of MDK and related genes in PBMC bulk gene expression data, with an AUC = 0.86.

In this analysis, we employed an approach to calculate the MDK score (referred to as MK score) in lung tissue by determining the mean expression levels of MDK-related genes. The set of MDK-related genes included MDK, TSPAN1, SDC1, SDC2, SDC4, protein tyrosine phosphatase receptor type Z1 (PTPRZ1), ITGA4, ITGA6, ITGB1, low-density lipoprotein receptor-related protein-1 (LRP1), NCL, and anaplastic lymphoma kinase (ALK). To explore the relationship between the MK score and clinical indicators, we utilized the GSE47460 dataset. Pearman’s correlation analysis revealed a moderate correlation between the MK score and clinical indicators, which included −0.466 for DLCO, −0.315 and −0.321 for pre- and post-FEV1, and -0.394 and −0.418 for pre- and post-FVC (Figure 6A). These findings support the feasibility of employing MDK-related genes as potential markers of IPF.

Moreover, we employed machine learning techniques to develop predictive models for the identification of IPF using three lung tissue bulk gene expression datasets and three PBMC bulk gene expression datasets. Prior to model development, we conducted rigorous quality checks and performed necessary data preprocessing on the lung tissue datasets. We utilized 316 samples for training and testing purposes, with an 8:2 ratio, and selected 78 independent validation samples. Among the various models (support vector machine, Adaboost, and random forest) after five-fold cross-validation and grid search, the support vector machine (SVM) model exhibited the highest accuracy of 0.838 in the test dataset. Importantly, the independent validation dataset demonstrated an accuracy of 0.821. The AUC of 0.94 and 0.86 for test and validate datasets (Figures 6B, C). Regarding the PBMC datasets, we performed a random split of the samples into training (227 samples) and testing datasets (56 samples) with an 8:2 ratio. The SVM model yielded an AUC of 0.86, with precision rates of 0.857 for IPF patients and 0.6 for the control group (Figure 6D). These findings suggest that MDK may serve as a potential marker gene for IPF diagnosis, highlighting its significant role in the context of IPF.

## 3 Methods

### 3.1 Data acquisition

The datasets utilized in this study were obtained from the Gene Expression Omnibus (GEO, https://www.ncbi.nlm.nih.gov/geo/). The datasets included the GSE47460, GSE110147, GSE134692, GSE48149, and GSE53845 gene expression datasets from lung samples (DePianto et al., 2015; Anathy et al., 2018; Cecchini et al., 2018; Sivakumar et al., 2019; Renaud et al., 2020), GSE135893 single-cell RNA sequencing dataset from lung samples (Habermann et al., 2020b), and GSE132607, GSE28042, as well as GSE38958 (Herazo-Maya et al., 2013; Huang et al., 2014; 2021) gene expression datasets from PBMC samples. A total of 365 IPF lung samples and 155 normal lung tissue bulk gene expression data, 184 IPF and 99 normal PBMC bulk gene expression data, as well as 12 IPF lung and 10 normal lung single-cell RNA sequencing data, were analyzed. Further details regarding the dataset can be found in Supplementary Table S6.

### 3.2 The process of bulk gene expression datasets

For each bulk dataset of lung samples, we first checked the quality of samples by measuring the distribution of relative log expression (RLE). Assuming the majority of expressed genes are not differentially expressed, the RLE values should generally be centered around 0 and spread within a limited range (Gregory Alvord et al., 2007). As shown in Supplementary Figure S4, most of the samples have RLE centered around 0 and spread within a small range (Supplementary Figure S4A, S4B). Then we performed differential analysis using the limma package for expression chips or EdgeR package for bulk gene expression data by selecting genes with absolute log2 fold change (|log2FC|) >1 and adjusted *p* < 0.05 as the differentially expressed genes in each dataset (Ritchie et al., 2015; Zhu et al., 2021). We subsequently selected genes with consistently up- or downregulated in at least two datasets and no opposite differential expression in other datasets like DEGs. Using the clusterProfiler package (Yu et al., 2012), we conducted pathway enrichment analysis of GO (Carbon et al., 2021), KEGG (Kanehisa et al., 2021), and Reactome (Gillespie et al., 2022) for the differentially expressed genes, using adjusted *p*-value <0.05 as the screening criteria to obtain relevant gene pathways. Additionally, we carried out PPI analysis of the selected differentially expressed genes through the protein network interaction database STRING (https://string-db.org/), which was then imported into the cytoscape 3.7.2 software (Shannon et al., 2003) and identified key gene modules using the MCODE plug-in. We used the haircut method with a node score of 0.2 and selected nodes with a degree of more than 2, maximum depth of 100, and k-core of 2 to discover relevant gene clusters.

### 3.3 The process of single-cell RNA sequencing

#### 3.3.1 Quality check

In this study, we utilized single-cell RNA sequencing analysis by R (version 3.6.0) and Seurat (version 4.0.1) (Stuart et al., 2019; Qiu et al., 2023). We first used emptyDrops method from Seurat to calculate and select FDR less than 0.1 as threshold to replace the empty droplets. After that, we used the PercentageFeatureSet function to calculate the proportion of mitochondrial genes in the cells and replaced the dead cells deciding by a mitochondrial genes proportion more than 25% combined with identifying RNA values of less than 1000 (Supplementary Figure S4C). For each sample, the top 2000 variable feature genes were selected by using the FindVariableFeatures function from 27674 genes in each cell. The repeatedly present variable genes were selected by using the SelectIntegrationFeatures function. Then, the samples were integrated using the FindIntegrationAnchors and IntegrateData functions. These integration steps can align cell populations from different batches to correct for technical differences between datasets. Then the integrated data for all cells were further processed using the ScaleData, RunPCA, and RunUMAP functions. Cells were finally clustered with the FindNeighbors and FindClusters functions. The cells are generally clustered by cell types and not by sample or disease status (Supplementary Figures S5A–D).

#### 3.3.2 Cell annotation, double droplets removal, and DEG calling

To classify cells with high accuracy, we divided the cell annotation process into two steps. Firstly, cells are divided into four major kinds: immune cells (PTPRC+), stroma cells (PTPRC-, EPCAM-, PECAM1-), epithelial cells (EPCAM+), and endothelial cells (PECAM1+, CLDN5+, VWF+, CDH5+, NRP1+) (Supplementary Figure S5E). Then, for each major subtype cluster, we conducted the same preprocess steps as described above and extracted marker genes from the CellMarker2.0 database (Hu et al., 2023) and the classical published paper (Travaglini et al., 2020), which is constructing the cell atlas of human lungs to classify the specific cell clusters with affiliated verification from the SingleR package (Aran et al., 2019). Furthermore, to remove the cells expressing markers of different cell types, which is caused by the doublet cells, we used the DoubletFinder R package to calculate the possible multi-droplet and removed the doublet cells identified by the pk > 0.25 (McGinnis et al., 2019). For each cell type, we identified differentially expressed genes (DEGs) using the FindAllMarkers function from the Seurat package with the following settings: logfc.threshold = 0.25, min.pct = 0.1, only.pos = True and test.use = "wilcox".

#### 3.3.3 Trajectory analysis and switch gene analysis

The R package monocle2 was used to perform pseudo-time-based cell trajectory analysis (Qiu et al., 2017) of four main types, separately. With the result of the cell trajectory analysis, switch genes were identified using R package GeneSwitches (Cao et al., 2020). The switch genes, which may influence cell differentiation and transformation, were further enriched by the GO and KEGG pathways by way of the find_switch_pathway function with default parameters to reflect the pathway expression in the pseudo-time.

#### 3.3.4 Cell communication analysis

To analyze the cell-to-cell interactions in different major types, we used R package CellChat (Jin et al., 2021). The computeCommunProb function was used to identify related ligand-receptor pairs in the cell communication, while the computeCommunProbPathway function was used to calculate the expression of the pathways related to the cells. Besides, we used the compareInteractions function to find the disparity communication pathways and ligand-receptor pair in the IPF and control groups.

### 3.4 Filter DEGs related to clinical indexes

To investigate the genetic basis of clinical data in lung fibrosis, we obtained the GSE47460 dataset with clinical data and removed samples unrelated to lung fibrosis (Anathy et al., 2018). Using the prognostic data within this dataset, we selected DLCO, FEV1 pre/post, and FVC pre/post as prognostic indicators. We then conducted a Pearson’s correlation analysis between genes (which includes MDK and TSPAN1) and clinical data. Differentially expressed genes of moderate correlation (>0.4) with at least one clinical criterion were treated as clinically relevant.

Additionally, we selected genes related to intercellular communication with MDK expression (MDK, SDC1, SDC2, SDC4, PTPRZ1, ITGA4, ITGA6, ITGB1, LRP1, NCL, ALK, TSPAN1) and calculated their average expression levels, resulting in a score named MK score. The MK score was also subjected to Pearson’s correlation analysis with the prognostic indicators.

### 3.5 Machine learning model building to classify IPF with MDK-related genes

To evaluate the function of MDK-related communication genes in the IPF, we used bulk gene expression datasets GSE47460, GSE110147, and GSE48149 as the lung tissue group and bulk gene expression datasets GSE132607, GSE28042, and GSE38958 as the PBMC group to construct machine learning models separately. For GSE132607, we selected samples with the source name of “COMET-IPF_Baseline” to represent IPF patients. Quality control and preprocessing are performed for each dataset, and batch effects between different datasets are eliminated by the SVA package’s Combat function (Leek et al., 2012). The scikit-learn python package is used in the model construction, cross-validation, and result visualization in this section.

To be specific, the GSE47460 has two sub-datasets sequenced by different platforms. The sub-dataset sequenced by GPL 14550 was selected as the validation dataset. We integrated the sub-dataset sequenced by GPL6480 and two other datasets (GSE110147 and GSE48149) to construct the training and test datasets by correcting the batch effect using the SVA package’s Combat function (Leek et al., 2012). To train and testing the model, we randomly split the integrated datasets into a training part and test part with a ratio of 8:2. On the other hand, for the PBMC samples, we first integrated GSE132607, GSE28042, and GSE38958 datasets by correcting the batch effect using the SVA package’s Combat function. Then the integrated dataset was randomly split into a training part and testing part with a ratio of 8:2. We selected random forest (RF), support vector machine (SVM), and AdaBoost algorithms as our testing models. We used the GridSearchCV function to select the best parameters of the model and set the 5-fold cross-validation during the training process.

## 4 Discussion

IPF is a chronic and progressive lung disease that predominantly affects the elderly population and is characterized by thickening and scarring of lung tissue, leading to difficulty breathing. Despite being associated with high mortality rates, its etiology remains unclear. However, recent advances in sequencing technology and single-cell sequencing provide new possibilities for comprehensively analyzing IPF pathogenesis. In light of these developments, this study endeavors to execute a multi-dimensional interrogation of assorted sequencing data modalities with the objective of pinpointing key genes implicated in IPF pathogenesis that exhibit a strong correlation with established clinical indices of pulmonary function.

To investigate IPF pathogenesis, we utilized five datasets of bulk gene expression data as well as one single-cell RNA-sequencing dataset for comprehensive analysis. Analysis of the bulk gene expression dataset revealed that upregulated genes were primarily enriched in the ECM and cytokine-cytokine related pathways, whereas downregulated genes were enriched in the regulation of G protein-coupled receptors. These pathways have been previously reported to be associated with IPF pathogenesis (Chanda et al., 2019). To further excavate the function behind the genes, we calculated the correlation coefficient of clinical indexes and process gene switch analysis. Among the final selected 22 important genes, we identified that the MDK gene has the potential to regulate certain physiological processes in the epithelial cell of IPF.

The MDK gene encodes the midkine protein associated with cell growth, migration, and angiogenesis, and it has been identified as a key regulator of epithelial and endothelial cells (Filippou et al., 2020). In endothelial cells, the MDK signaling pathway occurs separately in IPF for lymphoid endothelial cells as ligand cells and vein cells as receptors. The main differences were concentrated in epithelial cells, club cells, and ciliated cells in IPF, which accounted for the majority of MK signaling, while AT2 cells are major components of the control group. Coincidentally, the expression strength of the MDK signaling pathway matched the proportion of epithelial cells in both the IPF and control group. Additionally, switch gene analysis on the trajectory of AT2 cells indicated that MDK may be involved in the development of AT2 cells. Further research and analysis found that the pathway focused on communication with MDK as a ligand, with NCL, SDC1, and SDC4 acting as receptors. These genes have been shown to be involved in the EMT process.

EMT is critical factor considered to be involved in the pathogenesis of pulmonary fibrosis, leading to changes in the balance and communication between lung cell groups, and contributing to the development of IPF (Liu et al., 2019). Although there are some works that reveal the role of MDK in the EMT process, most of them are associated with physiological processes involved in organ formation during embryogenesis. In our study, by screening differentially expressed genes and analyzing their relation to prognostic indicators, we found that MDK regulates EMT processes by communicating with SDC1, SDC2, SDC4, NCL, and TSPAN1 in IPF patients. Notably, it was previously reported that the MDK gene has a certain effect on the TGFβ signaling pathway, which has the ability to induce the development of EMT, enabling epithelial cells to acquire a mesenchymal phenotype. In addition to MDK, genes related to cell communication have also been shown to affect the development of the TGFβ signaling pathway. Our results potentially suggested that extrabronchial secretory cells known as club cells may elicit TGFβ signaling by secreting MDK protein and binding to the ligand gene on AT2 cell surfaces. This stimulation leads to induce of EMT processes (Ichihashi et al., 2016; Liu et al., 2019; 2020; Thatikonda et al., 2023), thereby facilitating transformation of epithelial cells in IPF patients and contributing to the progression of pulmonary fibrosis.

In a noteworthy development, validation of the hypothesis was accomplished by demonstrating a correlation between MDK gene expression, the MK score computed utilizing these genes, and numerous clinical indicators. Furthermore, the IPF machine learning classification model exhibited high accuracy in both lung tissue samples (AUC = 0.94 for test dataset and AUC = 0.86 for validate dataset) and PBMC samples (AUC = 0.86). For comparative purposes, White employed logistic regression to uncover biomarkers in the blood of IPF patients, utilizing the OPN, SP-D, and MMP-7 genes for IPF patient prediction and achieving an AUC of 0.709 (White et al., 2016). Ley et al. reported an AUC of 0.76 using cCK18 to differentiate IPF from HP/NSIP (Ley et al., 2014). The elevated accuracy of the classification model in this investigation serves to bolster the evidence, supporting the substantial influence of MDK and its related communication in the pathogenesis of IPF.

Studies have shown that administration of bleomycin in mice has been shown to increase the expression of MDK in lung tissue, while the lung tissue of MDK gene knockout mice exhibited decreased expression of fibrosis markers such as collagen, α-SMA, TNF-α, and TGF-β. This suggests the importance of MDK in the inflammatory response and fibrosis process (Misa et al., 2017). Furthermore, studies by Horiba et al. (Horiba et al., 2000) have demonstrated that MDK can enhance the recruitment of inflammatory cells, which may be involved in promoting lung fibrosis. Zhang et al. (Zhang et al., 2015) have found that MDK plays a critical role in the mechanical stress-induced EMT spectrum in human lung epithelial cells. The absence of MDK weakened these EMT features. This indicates that MDK may promote lung fibrosis by interacting with Notch2 and activating angiotensin-converting enzyme (ACE) expression. Additionally, the research by Xu et al. (Xu et al., 2021) has revealed that inhibiting MDK can improve lung injury induced by sepsis through the ACE/Ang II pathway and the involvement of Notch 2. This further emphasizes the role of MDK and provides potential therapeutic value for MDK as a target. In summary, these studies suggest that MDK plays an important regulatory role in the pathogenesis of lung fibrosis, including promoting inflammation and extracellular matrix deposition, participating in epithelial-mesenchymal transition, and modulating ACE expression. Further research will help to elucidate the exact role and mechanisms of MDK in the development of IPF, providing new directions for future therapeutic strategies.

In addition, we noticed the myofibroblasts and lipofibroblasts mostly occur in the IPF group (Supplementary Figure S5F), where communication are related to the collagen of ECM. Previous studies have shown that the peptides and glycoproteins in the ECM stimulate fibroblast growth and activation, exacerbating the degree of lung fibrosis (Tian et al., 2019). In our study, fibroblasts increased ECM synthesis by raising collagen-related communication in myofibroblasts and lipofibroblasts by way of switch DEGs such as IGF1 and SFRP1 (Blackstock et al., 2014; Wang, 2020).

Despite the interesting and noteworthy findings, several limitations should be noticed. Firstly, although the machine learning model achieved notable improvement in identifying IPF samples, the model may be further improved with larger and more balanced datasets. Secondly, in our analysis, MDK and its receptors are important for IPF development. However, further functional experiments and mechanical studies would better resolve the relationship between MDK signaling and IPF. Additionally, it is worth noting that our original data lacked comprehensive information of factors such as gender, age, comorbidities, and clinical manifestations. Therefore, conducting further analysis that incorporates these variables would yield a more nuanced understanding of the association between MDK and IPF, particularly in different clinical contexts and human characteristics.

In summary, we employed a comprehensive analysis utilizing single-cell datasets and multiple bulk gene expression datasets to identify clinically relevant DEGs associated with IPF pathogenesis. We also incorporated a detailed examination of MDK gene regulation mechanisms and constructed a machine learning model to identify IPF patients based on both lung tissue and PBMC samples. Our study provides valuable insights for future investigations into the regulatory processes underlying IPF.

## Data Availability

The datasets presented in this study can be found in online repositories. The names of the repository/repositories and accession number(s) can be found in the article/Supplementary Material.

## References

[B1] Anathy V. Lahue K. G. Chapman D. G. Chia S. B. Casey D. T. Aboushousha R. (2018). Reducing protein oxidation reverses lung fibrosis. Nat. Med. 24, 1128–1135. 10.1038/s41591-018-0090-y 29988126 PMC6204256

[B2] Aran D. Looney A. P. Liu L. Wu E. Fong V. Hsu A. (2019). Reference-based analysis of lung single-cell sequencing reveals a transitional profibrotic macrophage. Nat. Immunol. 20, 163–172. 10.1038/s41590-018-0276-y 30643263 PMC6340744

[B3] Blackstock C. D. Higashi Y. Sukhanov S. Shai S. Y. Stefanovic B. Tabony A. M. (2014). Insulin-like growth factor-1 increases synthesis of collagen type I via induction of the mRNA-binding protein LARP6 expression and binding to the 5′ stem-loop of COL1a1 and COL1a2 mRNA. J. Biol. Chem. 289, 7264–7274. 10.1074/jbc.M113.518951 24469459 PMC3953245

[B4] Cao E. Y. Ouyang J. F. Rackham O. J. L. (2020). GeneSwitches: ordering gene expression and functional events in single-cell experiments. Bioinformatics 36, 3273–3275. 10.1093/bioinformatics/btaa099 32058565

[B5] Carbon S. Douglass E. Good B. M. Unni D. R. Harris N. L. Mungall C. J. (2021). The gene ontology resource: enriching a GOld mine. Nucleic Acids Res. 49, D325–D334. 10.1093/nar/gkaa1113 33290552 PMC7779012

[B6] Cecchini M. J. Hosein K. Howlett C. J. Joseph M. Mura M. (2018). Comprehensive gene expression profiling identifies distinct and overlapping transcriptional profiles in non-specific interstitial pneumonia and idiopathic pulmonary fibrosis. Respir. Res. 19, 153. 10.1186/s12931-018-0857-1 30111332 PMC6094889

[B7] Chanda D. Otoupalova E. Smith S. R. Volckaert T. De Langhe S. P. Thannickal V. J. (2019). Developmental pathways in the pathogenesis of lung fibrosis. Mol. Asp. Med. 65, 56–69. 10.1016/j.mam.2018.08.004 PMC6374163 30130563

[B8] DePianto D. J. Chandriani S. Abbas A. R. Jia G. N’Diaye E. N. Caplazi P. (2015). Heterogeneous gene expression signatures correspond to distinct lung pathologies and biomarkers of disease severity in idiopathic pulmonary fibrosis. Thorax 70, 48–56. 10.1136/thoraxjnl-2013-204596 25217476 PMC4472447

[B9] Filippou P. S. Karagiannis G. S. Constantinidou A. (2020). Midkine (MDK) growth factor: a key player in cancer progression and a promising therapeutic target. Oncogene 39, 2040–2054. 10.1038/s41388-019-1124-8 31801970

[B10] Gillespie M. Jassal B. Stephan R. Milacic M. Rothfels K. Senff-Ribeiro A. (2022). The reactome pathway knowledgebase 2022. Nucleic Acids Res. 50, D687–D692. 10.1093/nar/gkab1028 34788843 PMC8689983

[B11] Gregory Alvord W. Roayaei J. A. Quiñones O. A. Schneider K. T. (2007). A microarray analysis for differential gene expression in the soybean genome using Bioconductor and R. Brief. Bioinform 8, 415–431. 10.1093/bib/bbm043 17906332

[B12] Habermann A. C. Gutierrez A. J. Bui L. T. Yahn S. L. Winters N. I. Calvi C. L. (2020a). Single-cell RNA sequencing reveals profibrotic roles of distinct epithelial and mesenchymal lineages in pulmonary fibrosis. 10.1126/sciadv.aba1972 PMC7439444 32832598

[B13] Habermann A. C. Gutierrez A. J. Bui L. T. Yahn S. L. Winters N. I. Calvi C. L. (2020b). Single-cell RNA sequencing reveals profibrotic roles of distinct epithelial and mesenchymal lineages in pulmonary fibrosis. Available at: https://www.science.org. 10.1126/sciadv.aba1972 PMC7439444 32832598

[B14] Hamanaka R. B. O’Leary E. M. Witt L. J. Tian Y. Gökalp G. A. Meliton A. Y. (2019). Glutamine metabolism is required for collagen protein synthesis in lung fibroblasts. Am. J. Respir. Cell Mol. Biol. 61, 597–606. 10.1165/rcmb.2019-0008OC 30973753 PMC6827066

[B15] Herazo-Maya J. D. Noth I. Duncan S. R. Kim S. H. Ma S. F. Tseng G. C. (2013). Peripheral blood mononuclear cell gene expression profiles predict poor outcome in idiopathic pulmonary fibrosis. Sci. Transl. Med. 5, 205ra136. 10.1126/scitranslmed.3005964 PMC4175518 24089408

[B16] Hernandez D. M. Kang J. H. Choudhury M. Andrianifahanana M. Yin X. Limper A. H. (2020). IPF pathogenesis is dependent upon TGFβ induction of IGF-1. FASEB J. 34, 5363–5388. 10.1096/fj.201901719RR 32067272 PMC7136152

[B17] Horiba M. Kadomatsu K. Nakamura E. Muramatsu H. Ikematsu S. Sakuma S. (2000). Neointima formation in a restenosis model is suppressed in midkine-deficient mice. J. Clin. Investigation 105, 489–495. 10.1172/JCI7208 PMC289157 10683378

[B18] Hu C. Li T. Xu Y. Zhang X. Li F. Bai J. (2023). CellMarker 2.0: an updated database of manually curated cell markers in human/mouse and web tools based on scRNA-seq data. Nucleic Acids Res. 51, D870–D876. 10.1093/nar/gkac947 36300619 PMC9825416

[B19] Huang L. S. Mathew B. Li H. Zhao Y. Ma S. F. Noth I. (2014). The mitochondrial cardiolipin remodeling enzyme lysocardiolipin acyltransferase is a novel target in pulmonary fibrosis. Am. J. Respir. Crit. Care Med. 189, 1402–1415. 10.1164/rccm.201310-1917OC 24779708 PMC4098083

[B20] Huang Y. Oldham J. M. Ma S. F. Unterman A. Liao S. Y. Barros A. J. (2021). Blood transcriptomics predicts progression of pulmonary fibrosis and associated natural killer cells. Am. J. Respir. Crit. Care Med. 204, 197–208. 10.1164/rccm.202008-3093OC 33689671 PMC8650792

[B21] Ichihashi Y. T. Yamaoka T. Ohmori T. Ohba M. (2016). Up-regulation of Syndecan-4 contributes to TGF-β1-induced epithelial to mesenchymal transition in lung adenocarcinoma A549 cells. Biochem. Biophys. Rep. 5, 1–7. 10.1016/j.bbrep.2015.11.021 28955801 PMC5600357

[B22] Jin S. Guerrero-Juarez C. F. Zhang L. Chang I. Ramos R. Kuan C. H. (2021). Inference and analysis of cell-cell communication using CellChat. Nat. Commun. 12, 1088. 10.1038/s41467-021-21246-9 33597522 PMC7889871

[B23] Kanehisa M. Furumichi M. Sato Y. Ishiguro-Watanabe M. Tanabe M. (2021). KEGG: integrating viruses and cellular organisms. Nucleic Acids Res. 49, D545–D551. 10.1093/nar/gkaa970 33125081 PMC7779016

[B24] Kobayashi Y. Tata A. Konkimalla A. Katsura H. Lee R. F. Ou J. (2020). Persistence of a regeneration-associated, transitional alveolar epithelial cell state in pulmonary fibrosis. Nat. Cell Biol. 22, 934–946. 10.1038/s41556-020-0542-8 32661339 PMC7461628

[B25] Lee J. W. Chun W. Lee H. J. Min J. H. Kim S. M. Seo J. Y. (2021). The role of macrophages in the development of acute and chronic inflammatory lung diseases. Cells 10, 897. 10.3390/cells10040897 33919784 PMC8070705

[B26] Leek J. T. Johnson W. E. Parker H. S. Jaffe A. E. Storey J. D. (2012). The SVA package for removing batch effects and other unwanted variation in high-throughput experiments. Bioinformatics 28, 882–883. 10.1093/bioinformatics/bts034 22257669 PMC3307112

[B27] Ley B. Brown K. K. Collard H. R. (2014). Molecular biomarkers in idiopathic pulmonary fibrosis. Am. J. Physiol. Lung Cell Mol. Physiol. 307, 681–691. 10.1152/ajplung.00014.2014 PMC4280147 25260757

[B28] Lin X. Barravecchia M. Matthew Kottmann R. Sime P. Dean D. A. (2019). Caveolin-1 gene therapy inhibits inflammasome activation to protect from bleomycin-induced pulmonary fibrosis. Sci. Rep. 9, 19643. 10.1038/s41598-019-55819-y 31873099 PMC6928213

[B29] Liu G. Wang Y. Yang L. Zou B. Gao S. Song Z. (2019). Tetraspanin 1 as a mediator of fibrosis inhibits EMT process and Smad2/3 and beta-catenin pathway in human pulmonary fibrosis. J. Cell Mol. Med. 23, 3583–3596. 10.1111/jcmm.14258 30869194 PMC6484435

[B30] Liu Z. Jin H. Yang S. Cao H. Zhang Z. Wen B. (2020). SDC1 knockdown induces epithelial–mesenchymal transition and invasion of gallbladder cancer cells via the ERK/Snail pathway. J. Int. Med. Res. 48, 300060520947883. 10.1177/0300060520947883 32812461 PMC7441293

[B31] Martinez F. J. Collard H. R. Pardo A. Raghu G. Richeldi L. Selman M. (2017). Idiopathic pulmonary fibrosis. Nat. Rev. Dis. Prim. 3, 17074. 10.1038/nrdp.2017.74 29052582

[B32] McGinnis C. S. Murrow L. M. Gartner Z. J. (2019). DoubletFinder: doublet detection in single-cell RNA sequencing data using artificial nearest neighbors. Cell Syst. 8, 329–337. 10.1016/j.cels.2019.03.003 30954475 PMC6853612

[B33] Misa K. Tanino Y. Wang X. Nikaido T. Kikuchi M. Sato Y. (2017). Involvement of midkine in the development of pulmonary fibrosis. Physiol. Rep. 5, e13383. 10.14814/phy2.13383 28811360 PMC5582267

[B34] Morse C. Tabib T. Sembrat J. Buschur K. L. Bittar H. T. Valenzi E. (2019). Proliferating SPP1/MERTK-expressing macrophages in idiopathic pulmonary fibrosis. Eur. Respir. J. 54, 1802441. 10.1183/13993003.02441-2018 31221805 PMC8025672

[B35] Peng L. Wen L. Shi Q. F. Gao F. Huang B. Meng J. (2020). Scutellarin ameliorates pulmonary fibrosis through inhibiting NF-κB/NLRP3-mediated epithelial–mesenchymal transition and inflammation. Cell Death Dis. 11, 978. 10.1038/s41419-020-03178-2 33188176 PMC7666141

[B36] Qiu X. Mao Q. Tang Y. Wang L. Chawla R. Pliner H. A. (2017). Reversed graph embedding resolves complex single-cell trajectories. Nat. Methods 14, 979–982. 10.1038/nmeth.4402 28825705 PMC5764547

[B37] Qiu Y. Mo C. Xu S. Chen L. Ye W. Kang Y. (2023). Research progress on perioperative blood-brain barrier damage and its potential mechanism. Front. Cell Dev. Biol. 11, 1174043. 10.3389/fcell.2023.1174043 37101615 PMC10124715

[B38] Renaud L. da Silveira W. A. Takamura N. Hardiman G. Feghali-Bostwick C. (2020). Prominence of IL6, IGF, TLR, and bioenergetics pathway perturbation in lung tissues of scleroderma patients with pulmonary fibrosis. Front. Immunol. 11, 383. 10.3389/fimmu.2020.00383 32210969 PMC7075854

[B39] Ritchie M. E. Phipson B. Wu D. Hu Y. Law C. W. Shi W. (2015). Limma powers differential expression analyses for RNA-sequencing and microarray studies. Nucleic Acids Res. 43, e47. 10.1093/nar/gkv007 25605792 PMC4402510

[B40] Shannon P. Markiel A. Ozier O. Baliga N. S. Wang J. T. Ramage D. (2003). Cytoscape: a software Environment for integrated models of biomolecular interaction networks. Genome Res. 13, 2498–2504. 10.1101/gr.1239303 14597658 PMC403769

[B41] Sivakumar P. Thompson J. R. Ammar R. Porteous M. McCoubrey C. Cantu E. (2019). RNA sequencing of transplant-stage idiopathic pulmonary fibrosis lung reveals unique pathway regulation. ERJ Open Res. 5, 00117-2019. 10.1183/23120541.00117-2019 31423451 PMC6689672

[B42] Stuart T. Butler A. Hoffman P. Hafemeister C. Papalexi E. Mauck W. M. (2019). Comprehensive integration of single-cell data. Cell 177, 1888–1902. 10.1016/j.cell.2019.05.031 31178118 PMC6687398

[B43] Tanner L. Single A. B. Bhongir R. K. V. Heusel M. Mohanty T. Karlsson C. A. Q. (2023). Small-molecule-mediated OGG1 inhibition attenuates pulmonary inflammation and lung fibrosis in a murine lung fibrosis model. Nat. Commun. 14, 643. 10.1038/s41467-023-36314-5 36746968 PMC9902543

[B44] Thatikonda S. Pooladanda V. Tokala R. Nagula S. Godugu C. (2023). Niclosamide inhibits epithelial-mesenchymal transition with apoptosis induction in BRAF/NRAS mutated metastatic melanoma cells. Toxicol. Vitro 89, 105579. 10.1016/j.tiv.2023.105579 36870549

[B45] Tian Y. Li H. Gao Y. Liu C. Qiu T. Wu H. (2019). Quantitative proteomic characterization of lung tissue in idiopathic pulmonary fibrosis. Clin. Proteomics 16, 6–11. 10.1186/s12014-019-9226-4 30774578 PMC6364390

[B46] Travaglini K. J. Nabhan A. N. Penland L. Sinha R. Gillich A. Sit R. V. (2020). A molecular cell atlas of the human lung from single-cell RNA sequencing. Nature 587, 619–625. 10.1038/s41586-020-2922-4 33208946 PMC7704697

[B47] Wang H. Liu Y. Liang X. Yang G. Liu Y. Li F. (2020). Effects of Secreted frizzled-related protein 1 on inhibiting cardiac remodeling. Eur. Rev. Med. Pharmacol. Sci. 24, 6270–6278. 10.26355/eurrev_202006_21525 32572894

[B48] White E. S. Xia M. Murray S. Dyal R. Flaherty C. M. Flaherty K. R. (2016). Plasma surfactant protein-D, matrix metalloproteinase-7, and osteopontin index distinguishes idiopathic pulmonary fibrosis from other idiopathic interstitial pneumonias. Am. J. Respir. Crit. Care Med. 194, 1242–1251. 10.1164/rccm.201505-0862OC 27149370 PMC5114439

[B49] Xu J. Y. Chang W. Sun Q. Peng F. Yang Y. (2021). Pulmonary midkine inhibition ameliorates sepsis induced lung injury. J. Transl. Med. 19, 91. 10.1186/s12967-021-02755-z 33639987 PMC7913048

[B50] Yu G. Wang L. G. Han Y. He Q. Y. (2012). ClusterProfiler: an R package for comparing biological themes among gene clusters. OMICS 16, 284–287. 10.1089/omi.2011.0118 22455463 PMC3339379

[B51] Zhang R. Pan Y. Fanelli V. Wu S. Luo A. A. Islam D. (2015). Mechanical stress and the induction of lung fibrosis via the midkine signaling pathway. Am. J. Respir. Crit. Care Med. 192, 315–323. 10.1164/rccm.201412-2326OC 25945397

[B52] Zhu G. Fang C. Mo C. Wang Y. Huang Y. Li J. (2021). Transcriptomic analysis of granulosa cell populations proximal and distal to the germinal disc of chicken preovulatory follicles. Sci. Rep. 11, 4683. 10.1038/s41598-021-84140-w 33633274 PMC7907084

